# Feeling Younger Every Day? Daily Activities and Subjective Age in the Health and Retirement Study

**DOI:** 10.1093/geroni/igaf122.2169

**Published:** 2025-12-31

**Authors:** Wassim Tarraf, Sangwoo Ahn, Kimson Johnson, Yeonwoo Kim, Kafayat Mahmoud, Samuel Van Vleet, Jiao Yu, Briana Mezuk

**Affiliations:** Wayne State University, Detroit, Michigan, United States; The Pennsylvania State University, University Park, Pennsylvania, United States; University of Michigan Institute for Social Research, Ann Arbor, Michigan, United States; The University of Texas at Arlington, Arlington, Texas, United States; Boston University, Boston, Massachusetts, United States; Miami University, Oxford, Ohio, United States; Yale University, New Haven, Connecticut, United States; University of Michigan, Ann Arbor, Michigan, United States

## Abstract

Subjective age (i.e., how old one “feels”) reflects beliefs about aging, both positive and negative, and is consequential to health and well-being. The mechanisms through which subjective age is linked to health are likely through age-salient social interactions and engagement in health promoting daily activities. This study examined the relationship between subjective age and activities in the prior 24 hours. We used data from n = 4,251 adults aged >50-years who completed the 2020 Health and Retirement Leave-Behind Questionnaire. Weighted generalized linear models (logistic and Poisson) tested whether, and to what extent, feeling younger than one’s age (i.e., discrepancy between chronological and subjective age) is associated with (a) engagement in 19 typical daily relevant activities (No/Yes) and (b) time spent doing these activities (No time to 7+hours). All models adjusted for demographics, number of chronic conditions (r = 0-8), and limitations in activities of daily living (r = 0-5). On average, people felt 8.7 years older (SD: +11.8) than their actual age. Feeling younger was positively associated with volunteering, walking, exercising, socializing, reading, and religious activities, as well as more time spent doing these activities. Feeling younger was also linked to increased time spent traveling, running errands, and seeking quiet time (e.g. meditating). Controlling for health burden and functional impairments, feeling younger is associated with greater engagement in, and time spent doing, cognitive, physical, and social activities, offering opportunities for scalable, community-based programming focused on these activities to promote health and well-being.

